# Welding Fume in the Western Australian Mining Industry: Impact of a Change to the Workplace Exposure Standard

**DOI:** 10.3390/ijerph22081238

**Published:** 2025-08-08

**Authors:** Matthew Oosthuizen, Kerry Staples, Adelle Liebenberg, Kiam Padamsey, Marcus Cattani, Andy McCarthy, Jacques Oosthuizen

**Affiliations:** 1 School of Medical and Health Sciences, Joondalup Campus, Edith Cowan University, Joondalup, WA 6027, Australia; m.oosthuizen@ecu.edu.au (M.O.); a.liebenberg@ecu.edu.au (A.L.); k.padamsey@ecu.edu.au (K.P.); m.cattani@ecu.edu.au (M.C.);; 2 Department of Health (Western Australia), Epidemiology Directorate, Perth, WA 6004, Australia

**Keywords:** welding fume, exposure assessment, sampling

## Abstract

The aim of this study was to analyse the Western Australian (WA) Safety Regulatory System (SRS) database to assess compliance of the WA mining sector regarding workers exposure to welding fumes and to identify trends over time. De-identified data analysed to assess the impact of reducing workplace exposure standards (WES) for general welding fumes on industry compliance. Historical trend analysis shows a shift from 100% compliance to 100% non-compliance, based on mean values and 95% confidence intervals, with exposure levels remaining consistent over time. These findings highlight the need for current, innovative engineering solutions, and raise questions about the validity of current sampling methods. Powered air-purifying respirators (PAPRs) integrated with welding helmets can reduce exposures by up to 99.96%, making their adoption as industry best practice critical, yet current sampling methodologies measure welding fume levels outside PAPRs, thus potentially misclassifying workers who are adequately protected as non-compliant. The sampling method is also influenced by other particulate matter present in the workplace that may be due to grinding or other dust generating activities in the vicinity of the welder. Lower WES values necessitate a review of exposure assessment and reporting methods to accurately reflect worker exposures.

## 1. Introduction

Welding fumes are a complex mixture of airborne particles, including metal oxides, silicates, and fluorides which can cause harm to those exposed [1]. Assessing these fumes poses significant challenges for occupational hygienists. The aerosol composition welders are exposed to is determined by several factors such as the welding process, including the type of welding rod and metals being welded, shielding gases (flux) used in the process, and any other contaminants that may be present on the metal such as external coatings [2]. In 2022, it was estimated that approximately 11 million people worked as welders globally, with an additional 110 million workers potentially exposed to welding fumes due to their proximity to welding activities [2]. This is a concern as occupational hygiene activities are generally focused on welders, thus neglecting a large cohort of non-welders that are also exposed. A recent study found that while only welders underwent personal exposure monitoring, non-welders working in the same factories had measurable levels of manganese in their blood [3]. Although the association between exposure and neurological effects in non-welders was not statistically significant, the findings suggest potential secondary exposure and highlight a clear gap in current monitoring practices [3].

In 2017, the International Agency for Research on Cancer (IARC) classified welding fumes as a human carcinogen. This decision was based on sufficient evidence from over 50 epidemiological studies linking welding fume exposure to increased cancer risk. The assessment found that workers exposed to welding fumes had a significantly higher risk of lung cancer than unexposed workers, with a pooled risk ratio of 1.29 (95% CI 1.20–1.39) [4]. Subsequent meta-analyses reinforced these findings, reporting a 48% increased risk of tracheal, bronchial, and lung cancers among exposed workers (RR 1.48, 95% CI 1.29–1.70) [1].

Beyond cancer risks, epidemiological studies have also demonstrated that exposure to welding fumes results in an increased risk of ischemic heart disease, acute myocardial infarction, increased blood pressure, chronic obstructive pulmonary disease (COPD), asthma and pneumonia [5,6,7]. The severity of lung injury, including inflammation, oxidative stress, genotoxic effects, and DNA damage, is determined by the dose, which is influenced by both concentration and exposure duration. This relationship further supports the causal link between welding fume exposure and respiratory disease [4]. Pregnant women have an elevated risk, with exposure to welding fumes resulting in an increase in pre-term and low-birth-weight babies [2].

A Korean study used multiple linear and logistic regression models to assess the link between cumulative welding fume exposure and lung function in a cohort of shipyard welders (*n* = 240) [8]. Mean welding fume concentrations for arc welding (2.7 mg/m^3^), tack welding (1.6 mg/m^3^), and cutting (1.2 mg/m^3^) were used to calculate cumulative welding fume exposures by multiplying the mean exposures for each similar exposure group (SEG) by the duration of employment, as assessed by occupational history. Workers were subsequently categorised as having either low, intermediate, or high exposure. The odds of being diagnosed with COPD were significantly elevated for both medium (OR 3.9; 95% CI 1.4–13.3) and high-exposure workers (OR 3.8; 95% CI 1.03–16.2) when compared to those deemed to be exposed to low levels. The authors confirmed an association between welding fumes and increased risk of COPD among this cohort [8]. A recent cross-sectional survey (*n* = 634) assessed workers self-reported exposure to welding fume and their use of fume control measures. The survey included welders recruited from various workplaces across Australia. Work in confined and restricted places was common (47% of respondents) and most did not have mechanical ventilation (81.1%) and the use of powered air-purifying respirators (PAPRs) was deemed to be poor at 38.8%. Exposure to metals was also high Cr VI (73.7%) and nickel (49.9%) [9]. In another Australian study [10], the effectiveness of three common control measures designed to reduce worker exposure to welding fume were evaluated. The three controls included local exhaust ventilation (LEV), powered air-purifying respirators (PAPRs) and on-gun extraction. All experimental conditions were assessed against welding fume levels with no controls. It was determined that LEV hood capture is likely to reduce welding fume exposures by a factor of up to 9, while the use of on-gun LEV reduced breathing zone welding fume concentrations by a factor of 12 and these findings are supportive of the findings of Lehnert et al. [11], who reported exposure reductions between 70% and 90% using on-torch extraction or caption hoods. Driscoll et al. [12] in 2025, reported that very little is known regarding welding fume exposures and control in Australia, and they conducted an exposure assessment at 20 Australian workplaces located in New South Wales. Personal (*n* = 67) and static (*n* = 46) samples were collected and further analysed for metal content. Personal samples were collected in the breathing zone, outside of respiratory protective equipment. It was found that total welding fume concentration from personal sampling exceeded 1 mg/m^3^ (a newly established workplace exposure standard). Furthermore, it was reported that very few workplaces had active ventilation, and most workplaces rely on natural ventilation from large workspaces, open doors and high ceilings. Respiratory protective equipment was sub-optimal, with approximately 25% of participants using no respiratory protection while welding. This study is the first comprehensive Australian assessment of concentrations of welding fume particulate and gases and of control measures. The findings suggest considerable scope for improvement regarding welding fume exposure controls.

In January 2024, the Australian workplace exposure standard (WES) for welding fumes (not otherwise classified) was reduced from 5 mg/m^3^ (repealed WES) to 1 mg/m^3^ (current WES) measured as the total inhalable fraction [13]. Although a significant component of welding fume is regarded as respirable, with particles less than 1 µm in aerodynamic diameter, inhalable aerosol sampling as described in Australian Standard 3640:2009 [14] is the prescribed method for collection of welding fume samples. For personal exposure assessment of welding fume, the sampling head should ideally be positioned inside the welding helmet [15], although this is often not the case.

Assessment of welding fumes usually consists of two phases. In Australia total welding fume (mg/m^3^, total mass) is measured as per Australian Standard 3640:2009, [14] followed by an analysis of the sample filter for individual contaminants (metals), that are detailed in the Safety Data Sheets (SDS) of the metal used during the welding process and for any coatings on welded surfaces, the welding rods, and other consumables used. Examples of these individual contaminants include (but are not limited to) nickel, chromium, lead, aluminium, and manganese [2,13,14]. Table 1 provides a summary of contaminants generally present in different types of welding fumes.

It should be noted that obtaining homogeneous exposure samples among welders is challenging due to several factors that introduce variability in welding fume exposure [5]. A multi-national study, found that welding fume exposures vary considerably across countries and occupational groups with differences in industrial settings, types of ventilation, welding processes, and materials all contributing to large variations in exposures which have implications for the evaluation of potential adverse effects associated with welding fume exposures [5,9,10,11,12,14]. Additionally, research indicates that variable concentrations of welding fumes can result from improper use of local exhaust ventilation systems, variations in sampler positioning, differences in time spent welding, welding positions, and the skill levels of welders, collectively these factors make it difficult to obtain homogeneous exposure samples in welding environments [14]. These studies underscore the inherent challenges in standardising exposure assessments among welders, necessitating tailored strategies to accurately evaluate and mitigate health risks associated with welding fume exposure.

## 2. Aim and Hypotheses

The aim of this study was to analyse the SRS database to assess compliance of the Western Australian (WA) mining sector regarding workers exposure to welding fumes and to identify trends over time.

It was hypothesised that:

1. Most welding fume samples previously classified as compliant (before the 2024 change) exceed the WES of 1 mg/m^3^ TWA.
2. Compliance with the 2024 general welding fume WES will assure compliance with the respective WESs of individual contaminants contained within the welding fume.

## 3. Methods

All inhalable particulate sample data that were identified in the SRS database as welding fume samples were extracted by staff employed by the WA Department of Energy, Mining, Industry Regulation and Safety (DEMIRS). The data spans a period of several decades. An MS-Excell spreadsheet that was de-identified and contained neither personal nor agency (company) specific information was provided to researchers. The data was stored in a secure, password-protected online system, in accordance with the Edith Cowan University (ECU) Data Management plan associated with ethics approval 2023-04914-Oosthuizen. All data was shift adjusted to accommodate the work rosters common to WA mining, generally 12 h shifts for multiple consecutive days.

This longitudinal dataset was analysed using R 4.1.0. All welding fume data was stratified by commodity type, job role and work location to account for potential exposure variations due to different mining environments. Descriptive and predictive statistics were generated from the cleaned dataset and the mean, lower, and upper 95% confidence limits (lower-LCL95%, and upper-UCL95%) were evaluated to assess compliance for various similar exposure groups (SEGs) to both the repealed and current WESs. To examine recent temporal trends, a zero-adjusted gamma model (ZAG) was used, with commodity as a covariate, to assess if there has been a significant change in the expected results over the period, and whether there are any differences between the covariates of location and occupation, which were assumed to be SEGs for the analysis. ZAG models effectively account for skewed data distributions and zero results commonly observed in exposure datasets.

## 4. Results

There were 10,843 welding fume samples captured in the de-identified database, collected between 1986 and 2024. Almost all 10,748 (99.1%) were obtained from surface occupations, with only 95 (0.9%) of the samples obtained from underground locations. The largest number of samples were from iron ore (3737) and gold (2236) mines, as shown in Table 2.

The most commonly occurring individual contaminants analysed were iron oxide (*n* = 5739), nickel (*n* = 5147), chromium (*n* = 5050), zinc (*n* = 4387), manganese (*n* = 4283), and copper (*n* =4251).

From a total of 4035 samples collected between 2018 and 2024, 1221 samples (30.2%) were compliant with the current WES (1 mg/m^3^), meaning 2814 samples (69.7%) were not (Table 3). The proportion of all samples exceeding the repealed and current WES, stratified by commodity is also shown in Table 3. Most industry types exceed the current WES, some by as much as 89.5%.

### 4.1. Welding Fume Exposure Trends: A Comparison of WESs

**Figure 1:**
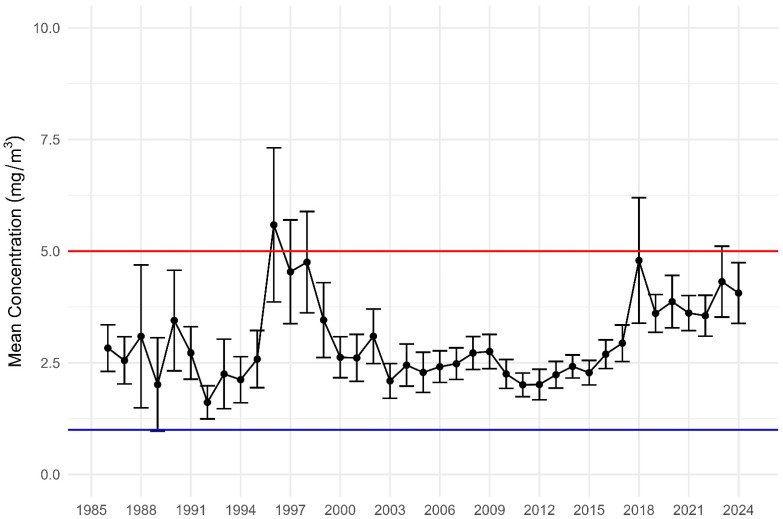
Mean Welding Fume Results, including LCL and UCL (1986 to 2024). Note: The red line represents the repealed WES and the blue line the current WES (2024).

To test the hypothesis that previously compliant samples will exceed the current WES, the mean and 95% confidence limits for welding fume samples were calculated. As shown in Figure 1, welding fume results have been relatively compliant with the repealed WES since 1985, except for the late 1990s and again in 2018 and 2023, a phenomenon that cannot be explained with the data available. As hypothesised, all mean and UCL95% levels exceed the current WES.

**Figure 2:**
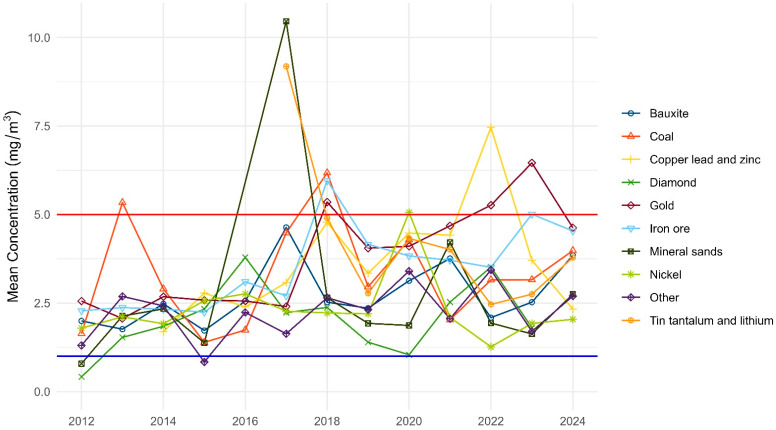
Mean welding fume results by commodity (2012 to 2024). Note only commodity types with 100 or more samples over the period have been included. Note: The red line represents the repealed WES and the blue line the current WES (2024).

The actual sample means by commodity for 2012 to 2024 are shown in Figure 2. No commodity types had a mean value below the WES in the past eight years. Over this period, the zero-adjusted gamma GLM modelling indicates the mean welding fume result is increasing by 1.5% per year (*p* = 0.02). The expected mean sample result for the iron ore industry in 2024 was 2.83 mg/m^3^ (95% CI 2.14 to 3.77 mg/m^3^), which is within the range of the repealed WES, but exceeds the current WES. When compared to the iron ore industry, the expected results for the diamond, mineral sands, nickel and other were significantly lower (*p* = 0.014, 0.011, <0.001, and 0.012, respectively). The remaining commodity types were not significantly different.

### 4.2. Welding Fume: A Proxy for Individual Contaminant Compliance

To test hypothesis two, the dataset was stratified to identify all samples that had also been analysed for additional contaminants (*n* = 6755). Of those, 3783 (34.9%) were compliant with the current WES of 1 mg/m^3^. Of these welding fume compliant samples, 9 (0.24%) exceed the WES for a different individual contaminant. These exceedances were recorded for vanadium (*n* = 2), lead (*n* = 2), nickel (*n* = 1), iron oxide (*n* = 2) and chromium VI (*n* = 2). Of those, only four exceedances, (2 × iron and 2 × lead) occurred within the past seven years, two each in 2017 and 2019.

## 5. Discussion

As hypothesised, historical data suggest that the industry is now deemed 100% non-compliant based on mean and 95% confidence intervals, this finding is supported by a recent (2025) Australian study by Driscoll et al. [12]. Furthermore, modelling data indicate that welding fume levels have in fact increased slightly over recent years, suggesting that existing control measures may be insufficient to meet the stricter standard. This stagnation implies that further reductions in exposure levels may not be achievable without substantial enhancements to current engineering controls and respiratory protection practices. An Australian study comparing the effectiveness of various control measures in reducing welding fume exposure was conducted recently [10]. The findings revealed that while local exhaust ventilation and on-gun fume extraction systems can significantly lower fume concentrations, their effectiveness varies depending on the welding process and environmental conditions, specifically worker positioning and duration of the LEV usage. The study also highlighted that PAPRs that are an integrated component of welding helmets provided the highest levels of protection against welding fumes, achieving exposure reductions of 99.96%, corresponding to an Effective Protection Factor (EPF) of 2600, which is considerably better than the protection factor of 50 as specified in AS/NZS 1715:2009 [11].

The hypothesis that compliance with the current welding fume WES would ensure compliance with all individual contaminants was strongly supported, with compliance at 99.6%. From a pragmatic industry and regulatory perspective, this level of compliance is likely sufficient. The necessity of analysing all constituent components of welding fumes warrants further investigation with due consideration of the toxicological critical effects of individual contaminants. It may be feasible to only require analysis of specific agents such as chromium (VI) and nickel compounds rather than all contaminants in the fume. A recent Australian study confirms this finding [12]. This hypothesis would benefit from further testing in prospective studies as well as by analysing other large historical datasets.

The issue of how protection provided by respiratory protection equipment, particularly that of PAPR, is factored into the assessment of worker exposure will need to be addressed by the regulators as more guidance is needed. The current approach, which does not consider the reduction in exposure when using high-efficiency respiratory protection, may lead to misleading compliance assessments. Future guidance should incorporate respirator-adjusted exposure limits to reflect real-world worker protection levels more accurately.

Secondary exposure among workers near welding operations remains an overlooked risk. Since these workers may not wear Respiratory Protective Equipment (RPE), they could face higher unprotected exposure than PAPR-equipped welders. Targeted exposure assessments should be conducted to quantify this risk and inform appropriate protective measures.

## 6. Limitations

A limitation of this study is that the exposure data captured in the SRS database does not currently collect data on the positioning of the sampling head, which may be placed either inside or outside the welding hood. This limitation reduces the accuracy of exposure assessments, particularly for PAPR users, as the protective effect of their respiratory equipment is not considered. The dataset, although large was developed for compliance monitoring and so there is a lack of specific hypothesis detail other than if levels were compliant with standards appropriate at the time, or not.

## 7. Conclusions

Data suggest that achieving compliance with the current WES would likely ensure compliance with other individual contaminants in welding fumes, as only 0.37% of compliant samples recorded exceedances in individual contaminants. However, further prospective studies specifically designed to test this hypothesis in different settings are warranted. This research should consider the various health impacts upon which the constituent exposure limits within the WES framework were based, such as the prevention of manganism, parkinsonism, and nasal cancer.

Achieving compliance with the current WES will require substantial improvements in engineering controls and strict adherence to the welding process codes of practice. This includes measures such as LEV, on-gun fume extraction systems, and RPE requirements. However, it is also likely that welding fume exposures have been historically over-estimated due to the sampling method used that would collect all inhalable particulate matter onto a filter, not just fume and this is an area of research that needs further investigation.

PAPRs integrated with welding helmets provide the most effective protection, with reductions in fume exposure by up to 99.96%. Current methods for interpreting sampling data need to be updated to accurately reflect actual worker exposures when RPE is used. This is important as in Australia new proposed exposure standards will allow for inclusion of protection factors in exposure assessment; however, there is a lack of guidance on requirements around the implementation of this practice. More research is needed to assess exposures if individuals working near welders as they may face higher exposure risks than welders using PAPRs.

This analysis provides valuable evidence to inform future regulatory strategies, exposure control priorities, and occupational health interventions.

## Figures and Tables

**Table 1:** Chemical properties of welding fume generated from various metals [15].

Metal Being Welded	Individual Contaminants (Associated Fumes)	May Also Contain
Mild steel	Iron, carbon, manganese, silicon, aluminium	Nickel, chromium, molybdenum, niobium, vanadium, boron
Stainless steel	Iron, chromium, nickel	Molybdenum, manganese, titanium
Aluminium	Aluminium, silicon, iron, copper, manganese, chromium, zinc, titanium	Gallium, vanadium, boron (wrought alloys), tin or lead (cast alloys)
Copper, Bronze, and Brass alloys	Copper, zinc, nickel, aluminium, tin, lead, silicon, iron	Manganese, tellurium, sulphur, chromium, cadmium, beryllium, silver, cobalt

**Table 2:** Welding fume samples (*n*) by commodity and location.

Commodity	Surface (*n*)	Underground (*n*)	Total (*n*)
**Bauxite**	1742	1	1743
**Chemical**	23	0	23
**Coal**	325	0	325
**Copper, lead and zinc**	214	6	220
**Diamond**	264	17	281
**Gold**	2318	60	2378
**Iron ore**	3897	0	3897
**Mineral sands**	227	0	227
**Nickel**	719	11	730
**Not specified**	56	0	56
**Other**	556	0	556
**Rare earth**	39	0	39
**Silica**	152	0	152
**Tin tantalum and lithium**	216	0	216
**Total**	10,748	95	10,843

**Table 3:** Welding fume samples exceeding the WES, stratified by commodity (2018–2024).

Commodity	Samples	Exceed Current	Exceed Current (%)	Exceed Repealed	Exceed Repealed (%)
**Iron ore**	1440	1056	73.3	333	23.1
**Gold**	1087	812	74.7	300	27.6
**Nickel**	349	164	47.0	34	9.7
**Bauxite**	266	166	62.4	47	17.7
**Other**	257	169	65.8	31	12.1
**Tin tantalum and lithium**	156	109	69.9	33	21.2
**Mineral sands**	141	78	55.3	17	12.1
**Copper lead and zinc**	122	97	79.5	30	24.6
**Coal**	75	51	68.0	18	24.0
**Not-specified**	41	30	73.2	10	24.4
**Rare earth**	38	34	89.5	3	7.9
**Silica**	29	24	82.8	6	20.7
**Diamond**	28	19	67.9	3	10.7
**Chemical**	6	5	83.3	0	-
**Total**	4035	2814	69.7	865	21.4

## Data Availability

All data supporting the findings of this study are contained within the confidential DEMIRS database of the Western Australian Government. The extracted de-identified data can be requested from the corresponding author.
